# Species interactions determine the spatial mortality patterns emerging in plant communities after extreme events

**DOI:** 10.1038/srep11229

**Published:** 2015-06-08

**Authors:** Jinbao Liao, Jan Bogaert, Ivan Nijs

**Affiliations:** 1 Research Group Plant and Vegetation Ecology, Department of Biology, University of Antwerp (Campus Drie Eiken), Universiteitsplein 1, B-2610 Wilrijk, Belgium; 2 Ministry of Education’s Key Laboratory of Poyang Lake Wetland and Watershed Research, Jiangxi Normal University, Ziyang Road 99, 330022 Nanchang, China; 3 Biodiversity and Landscape Unit, Université de Liège, Gembloux Agro Bio Tech, Passage des Déportés 2, 5030 Gembloux, Belgium

## Abstract

Gap disturbance is assumed to maintain species diversity by creating environmental heterogeneity. However, little is known about how interactions with neighbours, such as competition and facilitation, alter the emerging gap patterns after extreme events. Using a spatially explicit community model we demonstrate that negative interactions, especially intraspecific competition, greatly promote both average gap size and gap-size diversity relative to positive interspecific interaction. This suggests that competition would promote diversity maintenance but also increase community invasibility, as large gaps with a wide size variety provide more diverse niches for both local and exotic species. Under interspecific competition, both gap metrics interestingly increased with species richness, while they were reduced under intraspecific competition. Having a wider range of species interaction strengths led to a smaller average gap size only under intraspecific competition. Increasing conspecific clumping induced larger gaps with more variable sizes under intraspecific competition, in contrast to interspecific competition. Given the range of intraspecific clumping in real communities, models or experiments based on randomly synthesized communities may yield biased estimates of the opportunities for potential colonizers to fill gaps. Overall, our “static” model on gap formation offers perspectives to better predict recolonization opportunity and thus community secondary succession under extreme event regimes.

Gap formation is a common disturbance in many ecosystems. Because gaps of various sizes provide a wide range of regeneration niches for plant species with different traits^1^,^2^,^3^,^4^, the process of gap formation is considered to maintain species diversity^5^,^6^,^7^,^8^. For example, small gaps in which light cannot penetrate often accommodate shade-tolerant species, while large gaps are important for the establishment of shade-intolerant pioneer species^9^,^10^. Small openings are also more frequently occupied by clonal expansion of the surrounding plants, whereas seed establishment is more effective in large gaps^11^. Owing to more unused resources (e.g., light, water and soil nutrients), plant communities subjected to gap disturbance regimes may also be more susceptible to invasion^12^,^13^.

Identifying the mechanisms of gap formation is therefore key to understanding community structure and succession. Much of gap creation is driven by natural factors such as the lifespan of individuals, biotic disturbance (e.g., pest outbreaks, animal burrowing and herbivory) and abiotic disturbance (e.g., extreme drought, heatwaves and flooding)^11^,^14^,^15^,^16^. While these factors have been extensively examined, little is known about the way in which the original community structure prior to disturbance affects gap formation. Based on species-specific mortality responses to extreme perturbation, Li *et al.*^17^ have modelled that both increasing species richness and increasing species differences in mortality promote the average gap size and gap-size diversity, owing to increasing chances for gap mergence in more diverse systems. This is confirmed by observations^18^,^19^,^20^. The spatial pattern of plant species is another aspect of community structure that may influence the size diversity of gaps^21^. For example, randomly mixed communities generate randomly distributed small gaps with low size diversity^15^, while greater intraspecific clumping increases gap size and diversity^17^. However, these studies focused only on the importance of community characteristics for gap creation, ignoring species interactions.

Species interactions can be intra- or interspecific. The former are usually negative due to conspecific competition for the same resources^22^, while the latter can be negative (interspecific competition), positive (facilitation) or neutral. Evidence of negative interactions between species in nature abounds^23^. However, positive interspecific interactions are not rare, for example, facilitative non-trophic relationships are observed in many ecosystems^24^,^25^,^26^,^27^. Often, the positive interaction occurs because one species ameliorates a physical, physiological or trophic stress that otherwise compromises the fitness of a resource exploiter. Also during extreme events which drive much of the gap formation in plant communities, species interactions take place and – more importantly – influence the survival of individuals^28^,^29^,^30^,^31^. For example, individuals surrounded by fast-growing neighbours that consume more water may experience more stress and thus enhanced mortality during extreme drought^15^, whereas shaded individuals may be protected from excessive water loss during the same event^32^,^33^. In cold-climate ecosystems, larger individuals can protect smaller ones from freezing on cold nights by limiting net longwave radiation loss (similar to cloud cover)^25^,^34^. Interestingly, Greenlee and Callaway^34^ also found that bunchgrasses compete with perennial mustard for resource acquisition in a wet, cold year, while shade-driven facilitation occurs between them in a dry, hot year by decreasing leaf temperature, transpiration losses, or evaporation of soil moisture. Positive interactions appear to be especially common in extreme environments^28^,^29^,^30^,^31^. Yet, how species interactions modify gap formation by altering plant mortality in communities subjected to extreme perturbation is poorly understood.

Here we construct a spatial lattice model of multispecies communities subjected to extreme events, in which we vary species richness, intraspecific aggregation and interaction strength. Two types of interspecific interactions, including positive inter(+,+) and negative inter(−,−), are considered in this model, as well as intraspecific competition intra(−,−) and neutral interaction (0,0). To simulate gap formation following an extreme event, we directly apply neighbour-dependent mortality probabilities to the individuals in a single step. With the model, we investigate how the emerging gap patterns are determined by the original structure of the community, and ultimately how the aforementioned interactions alter these patterns.

## Results

### Effects of species interactions on gap pattern at different richness levels

**Figure 1:**
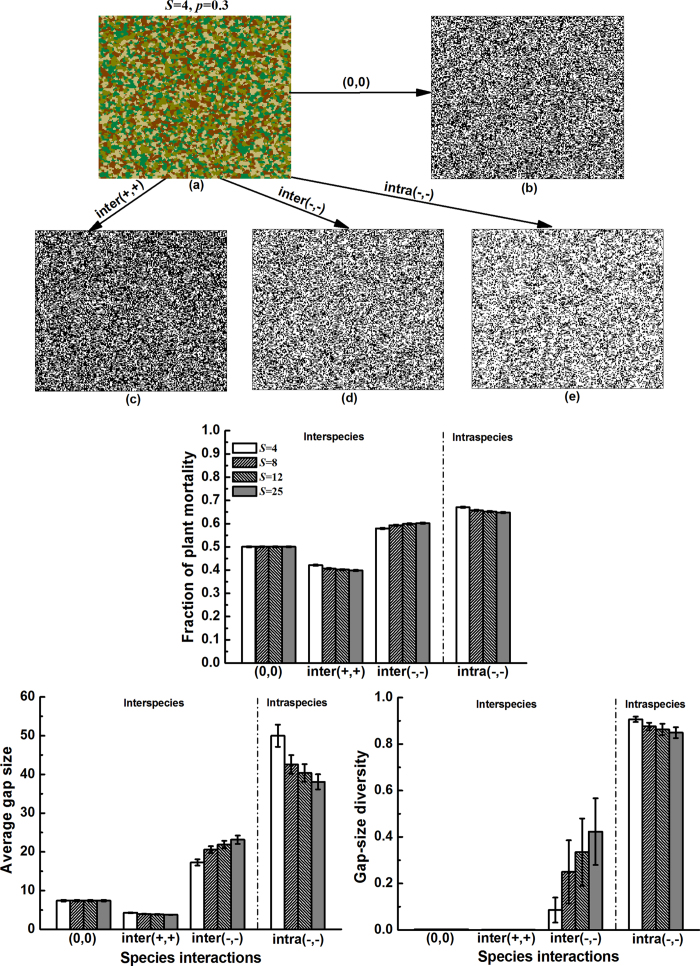
Top: illustration of gap creation displayed for species richness S = 4 under different interactions. (**a**) Community of 200 × 200 cells with four species (colours); (**b**) Gap pattern after assigning neighbour-dependent mortality under neutral interaction (0,0) (white - empty cells, black - occupied cells); (**c**) Gap pattern under positive interspecific interaction inter(+,+); (**d**) Gap pattern under negative interspecific interaction inter(−,−); (**e**) Gap pattern under negative intraspecific interaction intra(−,−). **Bottom: effect of species interactions on gap pattern under different species interactions**, characterized by fraction of overall mortality, average gap size and gap-size diversity (mean ± SD of 100 replicates) in communities with medium intraspecific clumping (*p* *=* 0.3), while varying species richness at *S* = 4, 8, 12, 25 (case 1 in Table 1).

The first set of simulations on the effects of interactions on gap pattern at different richness levels, demonstrated that negative interspecific interaction increased mortality while positive interaction decreased it (Fig. 1), according to expectation. Yet, intraspecific competition caused more deaths than interspecific competition, as intraspecific contacts outnumber interspecific contacts at the medium clumping level of the simulation. Species richness had minor effects on overall mortality, probably because species contact frequencies change only little with increasing richness. Following their impact on overall mortality, species interactions also strongly affected the gap metrics. Negative interactions, including inter(−,−) and intra(−,−), promoted both the average gap size and gap-size diversity compared to neutral interaction (0,0) (see illustration at *S* = 4 in Fig. 1). Since negative interactions enhance mortality of adjacent individuals, these effects obviously arise from gap mergence. This was strongest under intra(−,−), again because intraspecific contacts and therefore intraspecific competition dominated in this first set of simulations. In contrast, inter(+,+) decreased the average gap size, as it reduces the death rates of individuals located at the borders of their conspecific clumps, consequently blocking gap mergence between heterospecific clumps to some extent. However, this did not substantially modified gap-size diversity (Ψ ≈ 0 for both inter(+,+) and (0,0) in the right panel of Fig. 1), as the fairly low frequency of interspecific contacts at the medium clumping degree of the simulation only resulted in small gaps. Species richness also modulated the effects of species interaction on the gap pattern, but only for negative interactions. Under inter(−,−), it promoted gap size and gap-size diversity. The underlying mechanism is probably that interspecific contacts become more frequent when more species are present, which causes more deaths in neighbours belonging to different species, thus shaping larger gaps and consequently greater gap-size diversity (the latter because small gaps are always present). Conversely, species richness under intra(−,−) decreased both gap size and diversity, as intraspecific contacts are reduced.

### Impact of species difference in interaction strength on gap pattern

**Figure 2:**
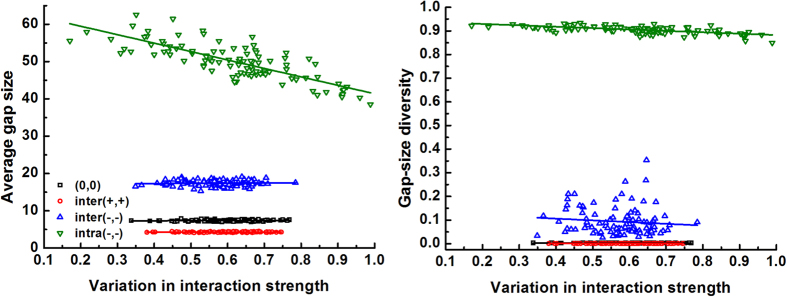
Effect of species variation in interaction strength 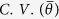 on gap metrics under different interactions (case 2 in Table 1, see Fig. 1 for interactions). For each interaction mode, 100 communities (keeping species richness at *S* *=* 4 and intraspecific clumping at *p* = 0.3) were assigned with randomly generated *θ*_*ij*_ (or *θ*_*ii*_) values around the mean |
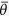
| = 0.125. Linear regressions were significant for each type of interaction (P < 0.01).

Next, we examined how the effects of species interactions on gap metrics are modified by species difference in interaction strength, in a community characterized by medium clumping (Fig. 2). Generally, interspecific differences in interaction strength (through expanding or reducing the range of *θ*_*ij*_ values) had little influence on gap size and gap-size diversity, regardless of interaction mode. Only enlarging the variation in intraspecific competition strength reduced the average gap size to a substantial extent. We assume that increasing *C.V*.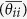 results in stronger intraspecific competition (greater *θ*_*ii*_) for some species, which may shape few large gaps, while other species which experience weaker intraspecific competition (lower *θ*_*ii*_) form many smaller gaps. This would ultimately diminish the average gap size. Similar to Fig. 1, negative species interactions, especially intraspecific competition, greatly promoted the average gap size and gap-size diversity.

### Effects of species interactions on gap formation modulated by intraspecific clumping

**Figure 3:**
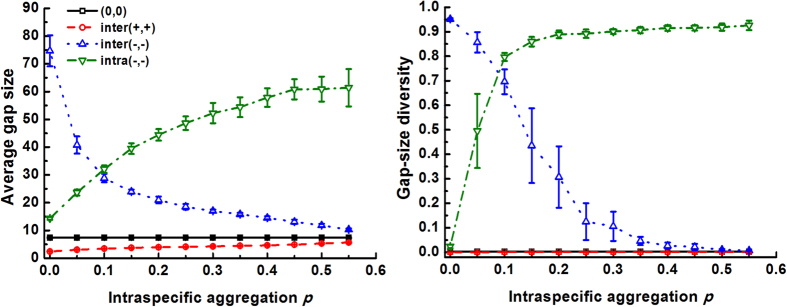
Effect of intraspecific clumping (*p*) on gap metrics (mean ± SD of 100 replicates) under different species interactions (case 3 in Table 1, see Fig. 1 for interactions).

Thirdly, we explored how intraspecific aggregation *p* modulates the effects of species interactions on gap metrics (Fig. 3). Similar to Figs. 1 and 2, inter(+,+) yielded a lower average gap size but similar gap-size diversity compared to neutral conditions (0,0), regardless of intraspecific clumping (all values were low). On the other hand, intraspecific aggregation largely altered the effects of inter- and intraspecific competition on the gap pattern. Under interspecific competition, the average gap size and gap-size diversity reached a maximum in randomly structured communities (i.e., *p* = 0), and declined exponentially with aggregation. This can be understood from the fact that randomly distributed communities have maximum interspecific contacts, so large numbers of individuals suffer from competition (note that over-dispersed communities, which were not simulated in this study, would probably yield larger gaps with higher gap-size diversity when facing extreme disturbance, as their intraspecific clumping is lower than randomly synthesized communities, inducing dominance of interspecific competition). In contrast, at *p* = 0, intraspecific contacts are minimized, hence the influence of intraspecific competition on gap formation is also minimal, resulting in the smallest average gap size and lowest gap-size diversity. With increasing clumping degree, both gap metrics increased rapidly as more and more individuals then experience intraspecific competition.

### Interactive impact of conspecific clumping and variation in interaction strength on gap pattern

**Figure 4:**
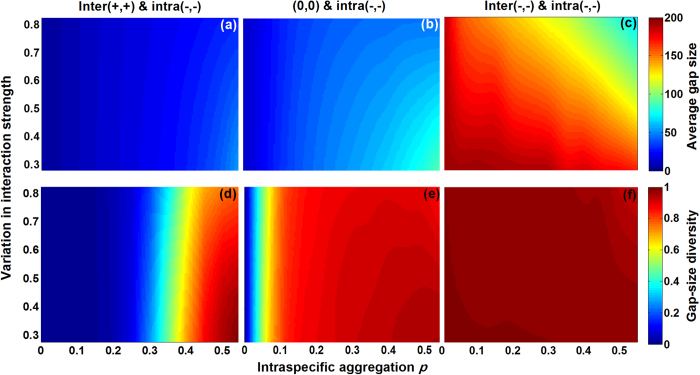
Additive effects of combined inter- and intraspecific interactions on gap metrics (mean of 100 replicates) (case 4 in Table 1, see Fig. 1 for interactions). Panels show colour maps of gap metrics as a function of intraspecific clumping *p* and species variation in interaction strength 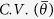 (with 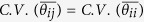 at *i* *≠* *j*).

In this final section we modelled the additive impacts of different interspecific interactions combined with intraspecific competition on gap metrics, at different clumping levels (*p*) and different levels of variation in both inter- and intraspecific interaction strength (with 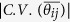 = 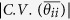 (*i* *≠* *j*)) (Fig. 4). Obviously, combining inter(−,−) and intra(−,−) greatly promoted gap size and gap-size diversity compared to other combinations (e.g., inter(+,+) & intra(−,−), and inter(0,0) & intra(−,−)), owing to much higher individual mortality rates. Likewise, neutral (0,0) associated with intra(−,−) created larger gaps and greater gap-size diversity than the combination of inter(+,+) & intra(−,−). However, both intraspecific clumping and variation in species interaction strength modified the patterns. Intraspecific aggregation promoted both gap metrics in the cases of inter(+,+) & intra(−,−) and inter(0,0) & intra(−,−). This can be understood from the fact that intraspecific competition progressively dominates the species interaction impacts on gap formation as conspecific clumping increases. Surprisingly, conspecific clumping largely reduced the average gap size in the combination of inter(−,−) & intra(−,−), especially at high 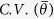. We assume that conspecific clumping decreases the probability of gap mergence between heterospecific clumps by reducing the interspecific contact frequency, thus decreasing the average gap size, even though a simultaneous increase of intraspecific encounters with *p* would compensate this to some extent (cf. Fig. 3). Gap-size diversity under the combined inter(−,−) & intra(−,−) interaction was always high because of the high death rates, which always yield sufficient numbers of large gaps regardless of the clumping degree. Focusing further on variation in both intra- and interspecific interaction strength, 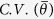 hardly influenced the average gap size and gap-size diversity in both inter(+,+) & intra(−,−) and inter(0,0) & intra(−,−). However, in the case of inter(−,−) & intra(−,−), increasing 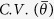 decreased the average gap size, especially at high clumping degree (Fig. 4c). As explained above, by enlarging the range of both *θ*_*ij*_ and *θ*_*ii*_, some species experience strong and others weak neighbouring competition, resulting in large gaps being outnumbered by small gaps. Combined, this decreases the average gap size. As mentioned above, gap-size diversity under combined inter(−,−) and intra(−,−) was always high (Fig. 4f).

## Discussion

In this study, we transformed initial species patterns of intact vegetation into gap spatial patterns by applying mortality probabilities in a single step. This “static” perspective makes the simulations most relevant for intense perturbations that occur within short time intervals, for example, extreme events such as severe drought and heatwaves, intense frost or flooding^14^,^16^,^17^. Since we did not focus on perturbations that gradually deteriorate the environment, gap dynamics was not incorporated in the simulations. In other words, factors like life span, reproductive success and seed dispersal were considered too slow relative to the impact of the perturbation. The applied mortality rates were neighbour-dependent, as species interactions alter intrinsic mortality upon exposure to extremes^28^,^29^,^30^,^31^. Based on empirical evidence^22^,^23^,^24^,^25^,^26^,^27^,^28^,^29^,^30^,^31^,^32^,^33^,^34^, the total impact of neighbour interactions on a target individual’s mortality in this study is assumed to be additive, as individuals surrounded by competitors may experience more stress and therefore enhanced mortality, while they may be protected when neighbours reduce environmental stress. Application of our model to types of pulse perturbation in which mortality may not involve significant interactions with neighbours (e.g., fire, lightning, animal burrowing, etc.) should be executed with neutral interaction (0,0). It should be stressed that the model only considers global disturbance by assuming that all individuals are disturbed in homogeneous landscapes, ignoring the spatial heterogeneity of possible localized disturbance. This reflects the global character of many extreme events, for example, extreme drought, heatwaves, flooding, etc.

By increasing the mortality probability of individuals, negative interactions but especially intraspecific competition, greatly increased the average gap size and gap-size diversity relative to neutral interaction (Fig. 1). This would promote the regeneration of resident species as well as newcomers, since potential gap-colonizers undergo reduced or no competition with local species in large gaps, and gaps of miscellaneous size provide a wide range of niches for species regeneration. In contrast, positive interspecific interaction decreased both the average gap size and gap-size diversity by blocking the mergence of gaps between heterospecific clumps, which may compromise diversity maintenance but enhance community resistance to invasion as fewer niches become available for both local and exotic species. Possible compromised diversity maintenance through this mechanism may offset the positive influence of facilitation on community richness by alleviating the impact of stress (e.g., water or nutrient limitation) or disturbance (e.g., extreme events) on species that would otherwise be intolerant to local conditions^25^,^26^,^35^.

We demonstrated that species interactions modify the opportunities for potential colonizers to establish following perturbation in different ways, depending on community characteristics. Increasing species richness strongly altered the effects of negative interactions on gap formation (Fig. 1). According to the modified random clusters principle for community generation^36^, increasing species richness promotes interspecific contact and consequently interspecific competition, thereby providing more chances for gap mergence between heterospecific clumps. This may be beneficial for potential colonizers filling these gaps as more diverse niches are provided. Conversely, when only considering intraspecific competition, the deceased conspecific contact associated with increasing richness leads to a decline in gap size and gap-size diversity, which may generate fewer niches available for both local and exotic species. Traditional theory asserts that species-rich communities are more difficult to be invaded than species-poor ones, owing to greater resource-use complementarity and consequently less unused resources^37^,^38^. However, viewed from a gap-disturbance perspective based on the current model, richness-invasibility relationships (which can be positive as well as negative) would largely be determined by the type of competition that dominates in the community (intra- or interspecific), provided increasing richness does not alter the mean and range of species intrinsic mortality^17^.

A second community characteristic – species difference in interaction strength – reduced the average gap size but only did so under intraspecific competition (Fig. 2). Whether local species can fill these smaller gaps may depend on gap location. Notably, species that are prone to strong intraspecific competition will be less successful in colonizing the small gaps close to their conspecifics, while they may occupy the centres of large gaps where intraspecific competition is avoided, or gaps within heterospecific clumps. Consequently, well-mixed species patterns may arise. Conversely, species experiencing weak intraspecific competition should be able to fill the gaps near conspecifics more easily, promoting their relative abundance and aggregation. From this perspective, having variation in intraspecific competition within the community may be key in regulating spatial pattern^39^. Interestingly, according to these inferences, competitors that are weak both intraspecifically and interspecifically should still be able to persist by maintaining their relative abundances and aggregation when recolonizing the gaps near their conspecifics, as also demonstrated by both theoretical^40^,^41^,^42^ and empirical work^21^,^22^,^43^,^44^,^45^,^46^.

Under intraspecific competition, conspecific clumping drastically enhanced the average gap size and gap-size diversity through gap mergence within conspecific clumps, but in the face of interspecific competition these metrics rapidly declined owing to less frequent contacts between species (Fig. 3). This suggests that intraspecific competition would promote species diversity by shaping more diverse niches when intraspecific aggregation increases. The inference of greater diversity in aggregated communities is also supported by traditional ecological theory (i.e., not considering gap formation), as species clumping reduces interspecific contact, thereby retarding competitive exclusion^47^. This is confirmed by numerous experiments with annual species^21^,^45^,^46^. On the other hand, conspecific aggregation may also promote community invasibility, as the increased gap size and gap-size diversity resulting from intraspecific competition can provide a diversity of niches for exotic species. Interestingly, another mechanism also supports this inference: if species are spatially correlated in clumps, then intraspecific competition prevails whereas interspecific competition is correspondingly weakened. This can lower resource use complementarity and thus resource uptake^44^,^48^, leaving more unused resources for newly establishing invaders. From a probabilistic perspective, multispecies communities start resembling monocultures as intraspecific aggregation increases, which can reduce the contact probabilities between resistant natives and exotic invaders, further weakening community resistance to invasion.

We simulated how species interactions modify gap patterns emerging in plant communities after extreme events. Analyzing the resulting gap patterns can estimate the spatial variation in light availability, which may help explain species coexistence especially in forest ecosystems based on the low light survival - high light growth trade-off and the heterogeneity in light availability created by canopy gaps, as confirmed by Gravel *et al.*^49^. Our findings also have implications for the interpretation of both experimental and model studies. For example, the fact that intraspecific clumping strongly modulates the effects of inter- and intraspecific competition on gap formation, suggests that experiments or models based on randomly structured communities may underestimate the colonization opportunities for both local and exotic species, given the wide range of intraspecific clumping observed in real communities. Understanding the interactive effects of species interactions and pre-disturbance community characteristics on gap formation can help explain observations from natural systems exposed to gap-disturbance regimes in general, and can support forecasting of community vulnerability to alien species, with gap patterns possibly serving as forewarning of species invasion. Overall, our modelling framework offers a basis for better prediction of recolonization opportunity and community secondary succession under extreme event regimes.

## Methods

### Spatially structured communities

We first simulate multispecies communities with a two-dimensional square lattice of size *L* × *L* = 200 × 200 cells (*L* is the length of the lattice). Each cell is occupied by an individual plant. Using the landscape generation software SIMMAP based on a modified random clusters method^36^,^50^, community patterns are produced by varying the following parameters: (1) species richness *S*, with the same population size of *L*^2^*/S* individuals attributed to each species; (2) intraspecific clumping degree *p* (0 ≤ *p* ≤ *p*_*c*_), which describes the frequency with which a nearest neighbour of a given individual belongs to the same species (following Hiebeler^51^), with the percolation threshold *p*_*c*_ = 0.5928 representing the maximum intraspecific aggregation under the default 4-neighbour principle^36^. Randomly structured communities have *p* = 0. Note that over-dispersed community patterns cannot be simulated using SIMMAP. Although they exist (e.g., some forests), over-dispersed communities are relatively rare in nature because of seed dispersal limitation, clonal growth or patchy environment^21^.

Without defining specific landscape processes (i.e., neutral landscape model), the SIMMAP software can generate the patchy and irregular plant communities that are typical in nature by fixing the above parameters^36^. The stochastic community assembly in SIMMAP can be justified by Hubbell’s neutral theory^52^ that natural communities can be recreated by only considering stochastic processes of birth, death and dispersal. As the simulations are generic, SIMMAP has been applied to a wide variety of vegetation types (e.g., temperate and tropical forest, temperate grassland and savanna) where plants largely cover the soil^53^,^54^,^55^.

### Gap formation

Next, the synthesized communities are hit by an extreme event, simulated by directly applying neighbour-dependent mortality rates to all individuals. This creates a gap pattern in a single step (see illustration in Fig. 1), representing the rapid catastrophic impact of extreme events whilst taking into account neighbour influences as described in the Introduction. Whether an individual of species *i* survives is determined by two factors: its intrinsic mortality and neighbouring interactions. The influence of intrinsic mortality on gap formation has been explored by Li *et al.*^17^. For this reason, and in order to allow the species interactions to both enhance and reduce mortality, we set the intrinsic mortality rate of all species to a medium value *m* = 0.5.

Following experimental results^56^, we assume that interaction is limited to nearest neighbours, while the total impact of neighbour interactions on a target individual’s death rate is assumed to be additive^21^,^57^. The neighbour influence on mortality is characterized by a direction (positive or negative) and a strength *θ*. The death rate of a target individual of species *i* at location *x* therefore equals


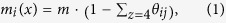


where species interactions can increase (negative interaction) or decrease (positive interaction) the mortality of a target individual (*j* → *i*). We use von Neumann neighbourship with each individual having four nearest neighbours (*z* = 4). The parameter *θ*_*ij*_ (0 ≤ |*θ*_*ij*_| ≤ 0.25) scales the degree to which a neighbouring species *j* decreases (*θ*_*ij*_ ≥ 0) or increases (*θ*_*ij*_ ≤ 0) the mortality of species *i*, and its absolute value |*θ*_*ij*_| represents the interaction strength. Species differences are represented by the coefficient of variation in species interaction strength 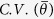, where 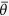 is the mean of all *θ*_*ij*_ values (differentiating between interspecific at *i* *≠* *j* and intraspecific at *i* *=* *j*). In the simulations, 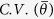 is varied by expanding or shrinking the range of |*θ*_*ij*_| values around the mean interaction strength 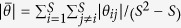 at *i≠j* (interspecific) or 
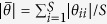
 at *i* *=* *j* (intraspecific) (Supplementary A), with a larger 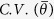 indicating a wider range of interaction strengths.

### Gap metrics

After gap formation, the FRAGSTATS 4.0 software^58^ is applied to recognize each gap based on the principle of 4-nearest neighbours. An empty cell formed by the death of an individual is treated as an isolated gap only if its four nearest neighbours are still occupied by survivors, otherwise it belongs to another gap. Two metrics are used to describe the gap pattern: the average gap size (i.e., the mean gap area based on cell counts) *ā* = 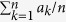 and the gap-size diversity 
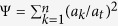
17,59, where *n* is the total number of gaps in the whole lattice, *a*_*k*_ the *k*-th gap size, and *a*_*t*_ *=* 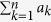 the total gap area. Both these metrics have ecological significance: (1) The average gap size *ā* indicates local colonization opportunity (invasion opportunity in case of exotic species). For example, large gaps can be colonized by a greater diversity of species^1^,^2^. (2) Gap-size diversity Ψ estimates the species range of potential colonizers filling these gaps, with larger Ψ indicating a wider species range. For example, species with different reproductive traits (e.g., clonal growth and seed dispersal) or shade-tolerance may prefer to colonize gaps of differing size^9^,^11^,^60^.

### Simulation cases

In the simulations, we focus on the impacts of intraspecific competition intra(−,−) and of two types of interspecific interactions (including inter(+,+) and inter(−,−)) on gap formation, by comparing with the case of neutral interaction (0,0) (i.e., control). Asymmetric interspecific interactions, including (+,0), (−,0) and (+/−), are also simulated, but have qualitatively similar effects on gap metrics as respectively inter(+,+), inter(−,−) and (0,0) (not shown). By varying community structure including species richness, intraspecific clumping and species difference, we model four cases as outlined below (see Table 1). The parameter 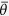 is kept constant in these simulations (effects of variation in 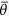 are shown in Supplementary B).

We first explore how species interactions change gap metrics at different richness levels (*S* = 4, 8, 12 and 25), while keeping intraspecific aggregation at *p* = 0.3 (medium) and species difference in interaction strength at 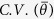 = 0.575 around the mean |
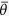
| = 0.125. At each richness level, four different interactions are considered: intra(−,−), inter(−,−) and inter(+,+), and control (0,0). For each type of interaction, we simulate 100 replicates to minimize stochastic errors, starting from different community patterns in each replicate, but with the same *S* and *p*. Species interaction strengths |*θ*_*ij*_| (or |*θ*_*ii*_|) are randomly generated at the same 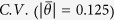 = 0.575. Ultimately, the means of these 100 replicates yield the average gap metrics.

Secondly, we test how the effects of species interactions on gap metrics are modified by species difference in interaction strength 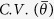. For each interaction mode, we generate 100 communities with the same intraspecific clumping *p* = 0.3 and richness *S* = 4, but different 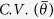 by randomly generating the values of *θ*_*ij*_ (or *θ*_*ii*_) around the mean |
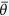
| = 0.125.

Next, we examine how intraspecific clumping modulates the effects of species interactions on gap metrics. Species richness is set to *S* = 4, and each case is explored with 100 replicates, similar to the first set of simulations.

Finally, we explore the additive effects of interspecific interaction and intraspecific competition on gap metrics at *S* = 4, while varying intraspecific clumping and species difference in interaction strength (with 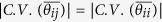 at *i* *≠* *j*). Also in this case we run 100 replicates for each combination, the means of which yield the average gap metrics. Based on these discrete mean values of gap metrics, a colour map is generated by interpolation.

## Additional Information

**How to cite this article**: Liao, J. *et al.* Species interactions determine the spatial mortality patterns emerging in plant communities after extreme events. *Sci. Rep.* **5**, 11229; doi: 10.1038/srep11229 (2015).

## Supplementary Material

Supplementary Information

## Figures and Tables

**Table 1:** **Four cases of gap simulation upon exposure to extreme events.**

**Simulation case (Figure)**	**Community structure**			**Species interaction type**
	**Species richness** ***S***	**Species difference** ***C.V.***( 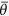 )	**Intraspecific clumping*****p***	
1	4,8,12,25	0.575	0.3	inter(−,−), inter(+,+), intra(−,−), (0,0)
2	4	Varied (random generation)	0.3	inter(−,−), inter(+,+), intra(−,−), (0,0)
3	4	0.575	0,0.05,∙∙∙,0.55	inter(−,−), inter(+,+), intra(−,−), (0,0)
4	4	0.275,0.325,∙∙∙,0.825	0,0.05,∙∙∙,0.55	intra(−,−) & inter(+,+), intra(−,−) & inter(−,−), (intra(−,−) & inter(0,0)

Species have the same intrinsic mortality rate *m* = 0.5, which species interactions increase or decrease by a maximum amount 0.5. Note that the 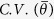 values in Fig. 4 were assigned to both inter- and intraspecific interactions at the same time, with |
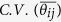
| = |
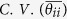
| at |
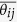
| = |
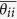
| = 0.125 (*i* *≠* *j*). Other parameters: mean interaction strength |
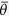
| = 0.125.
