# Supplementary material for: Species interactions determine the spatial mortality patterns emerging in plant communities after extreme events
Source: Sci Rep. 2015 Jun 8;5:11229. doi: 10.1038/srep11229 (PMC4459219; doi:10.1038/srep11229)
Supplement: Supplementary Information [file srep11229-s1.pdf]

Species interactions determine the spatial mortality patterns emerging in plant communities after extreme events

Jinbao Liao[*,a], Jan Bogaert[b], Ivan Nijs[a]

[a]Research Group Plant and Vegetation Ecology, Department of Biology, University of Antwerp (Campus Drie Eiken), Universiteitsplein 1, B-2610 Wilrijk, Belgium

[b]Biodiversity and Landscape Unit, Université de Liège, Gembloux Agro Bio Tech, Passage des Déportés 2, 5030 Gembloux, Belgium

[*]Corresponding author: jinbao.liao@uantwerpen.be

Tel.: +32 3 265 22 57;   Fax: +32 3 265 22 71

Supplementary Information

Supplementary A

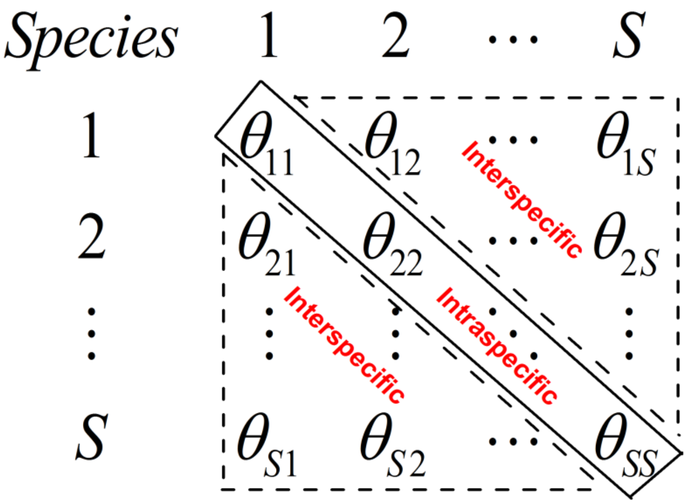

Figure A.1 Matrix of intra- and interspecific interaction, in which we separately calculated the average interspecific interaction strength $\left|\overline{\theta_{ij}}\right| = \sum_{i=1}^{S} \sum_{j \neq i}^{S} \left|\theta_{ij}\right| / \left(S^2 - S\right)$ at *i*≠*j* (mean of all θ_{ij} values in the two dashed triangles) and the average intraspecific interaction strength $\left|\overline{\theta_{ii}}\right| = \sum_{i=1}^{S} \left|\theta_{ii}\right| / S$ at *i*=*j* (mean of all θ_{ii} values in the solid rectangle), as well as their $C.V.(\overline{\theta_{ij}})$ and $C.V.(\overline{\theta_{ii}})$.

Supplementary B

Under different species interactions, we varied the parameter values of $\bar{\theta}$ (mean interaction strength) to analyze the sensitivity of gap metrics to the parameter (Figure B.1). Generally, gap metrics were more sensitive to variation in $\bar{\theta}$ when only negative interactions (i.e., species competition) were present, while they were insensitive to $\bar{\theta}$ under inter(+,+). An increase of $\bar{\theta}$ greatly enhanced gap size but only under intra(-,-). At the same time, gap-size diversity under intra(-,-) strongly increased beyond $\bar{\theta}$ >0.05, saturating at $\bar{\theta}$ =0.125. Interestingly, gap-size diversity under inter(-,-) was promoted substantially above $\bar{\theta}$=0.125, though average gap size was always small. Selecting the intermediate value $\bar{\theta}$=0.125 can thus represent the trends of gap patterns under different species interactions. Overall, varying $\bar{\theta}$ largely modified the negative interaction (especially intraspecific competition) effects on gap formation (gap metrics under other interactions were not influenced), but did not alter the general trends observed at the fixed $|\bar{\theta}|$=0.125 used throughout the paper.

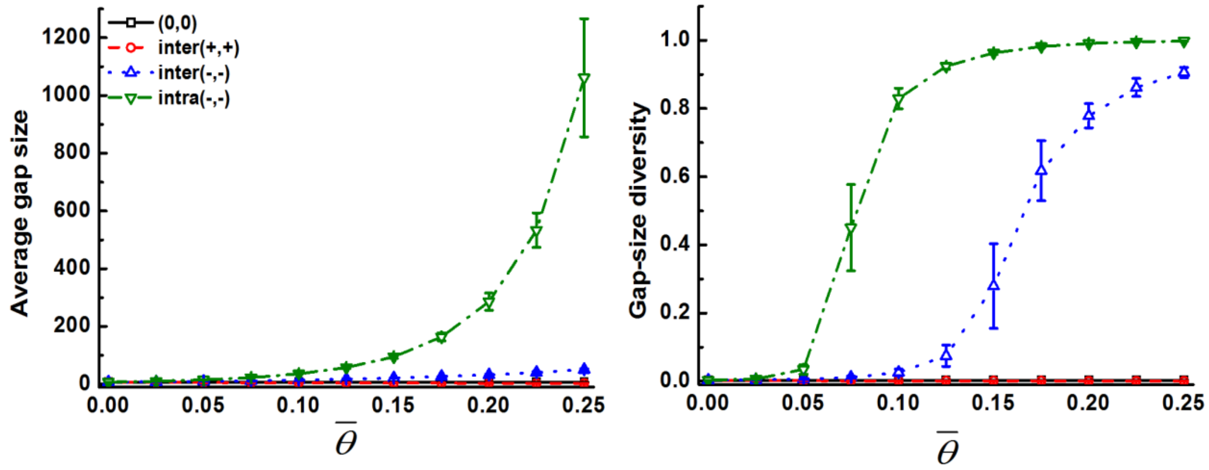

Figure B.1 Effect of variation in mean interaction strength (0≤$|\bar{\theta}|$≤0.25) on gap metrics (mean ± SD of 100 replicates) in communities with medium intraspecific clumping *p*=0.3 and species richness *S*=4. Other parameters: species intrinsic mortality *m*=0.5, maximum amount of species interactions influencing mortality also equals to 0.5, and species difference in interaction strength C.V.($\bar{\theta}$)=0.
